# Synthesis and Characterization of an Environmentally Friendly Phenol–Formaldehyde Resin Modified with Waste Plant Protein

**DOI:** 10.3390/polym15132975

**Published:** 2023-07-07

**Authors:** Hanyin Li, Sen Wang, Xiang Zhang, Hao Wu, Yujie Wang, Na Zhou, Zijie Zhao, Chao Wang, Xiaofan Zhang, Xian Wang, Cheng Li

**Affiliations:** 1 College of Forestry, Henan Agricultural University, Zhengzhou 450002, China; 2 College of Landscape Architecture and Art, Henan Agricultural University, Zhengzhou 450002, China; 3 Bureau of Agricultural and Rural Affairs, Luanchuan County, Luoyang 471500, China

**Keywords:** phenol–formaldehyde resin, soybean meal, soy-based adhesives, curing, plywood

## Abstract

To develop a lower-cost, excellent-performance, and environmentally friendly phenol–formaldehyde (PF) resin, soybean meal was used to modify PF resin, and soybean meal–phenol–formaldehyde (SMPF) resins were prepared. This reveals the effect of soybean meal on the structural, bonding, and curing properties of PF resin, which are very important for its applications in the wood industry. The resins’ physicochemical properties and curing performance were investigated, showing that SMPF resins have higher curing temperatures than PF resin. The Fourier transform infrared spectroscopy results indicated that a cross-linking reaction occurred between the amino groups of soybean protein and the hydroxymethyl phenol. Moreover, with the addition of soybean meal, the viscosity of SMPF increased while the gel time decreased. It is worth mentioning that SMPF-2 resin has favorable viscosity, short gel time, low curing temperature (135.78 °C), and high water resistance and bonding strength (1.01 MPa). Finally, all the plywoods bonded with SMPF resins have good water resistance and bonding strength, which could meet the standard (GB/T 17657-2013, type I) for plywood. The optimized SMPF resins showed the potential for application to partially replace PF resin in the wood industry.

## 1. Introduction

Phenol–formaldehyde (PF) resins have excellent properties, such as water resistance, bonding performance, and environmental stability [1,2], and have been widely used as cross-linked agents in wood-based panels and composites. However, their high cost and toxicity restrict their broader applications. To overcome these shortcomings, many researchers have focused on modifying PF resin using environmental and economic resources [3,4]. These materials have included urea [5,6,7], lignin [8,9], tannin [10,11], carbohydrate [12], bio-oil [13,14], soybean meal [15,16], and so on [17,18].

Soybean meal is a major byproduct of soybean oil processing. The use of soybean meal to prepare soy-based adhesives has a long history. Soy-based adhesives were very popular in the late 1920s and 1930s [19,20]. However, their poor water resistance and biological stability limited their application [21,22,23]. In the 1960s, soy-based adhesives were nearly completely replaced by petroleum-based adhesives in the wood industry [24]. Much time in recent decades has been devoted to enhancing soy-based adhesive properties (such as water resistance and bonding strength) [25,26,27]. These efforts can be classified into three strategies: denaturing modification, cross-linking modification, and protein molecular modification [25,27,28,29]. Cross-linking modification has been proven to be the most efficient method to enhance the comprehensive performance of soy-based adhesives [30]. Based on previous reports, many cross-linkers have been successfully used, such as epoxy resin, melamine–urea–formaldehyde resin, rubber, hyperbranched polymer, PF resin, and so on [25,31,32]. Among them, PF has aroused researchers’ interest [30]. Some studies have found that the hydroxymethyl groups of PF resin can react with the amino groups of soy protein under basic conditions [21,33,34]. However, it is still hard to meet the requirements of outdoor wood-based products by using these methods to modify soy-based adhesives. Many studies have also been devoted to reducing the cost and improving the environmental properties of PF adhesives with soybean meal, a lower-cost, environmentally friendly, and renewable material for partially replacing phenol and formaldehyde in wood adhesives [24]. Many studies have showed that the soybean meal-modified PF adhesives have good physical properties for bonding wood panels [21,29]. However, the properties of water resistance were decreased. In addition, some research has focused on the effect of soybean meal on the structural, bonding, and curing properties of PF resin. These properties are very important for its applications in the wood industry.

In our previous research, it was found that soy protein isolate could promote the curing of phenolic resin at low temperatures. In fact, soy protein isolate is highly cost as a modifier of phenolic resin. To obtain a low-cost, excellent-performance, and environmentally friendly protein-based phenolic resin which was applied in the wood industry, in this study, soybean meal–phenol–formaldehyde (SMPF) resins with varying contents of soybean meal were prepared and applied to plywood. The thermal performances of SMPF were detected using differential scanning calorimetry (DSC) and thermogravimetric (TG) analysis. At the same time, the physical properties of SMPF resin were also investigated.

## 2. Materials and Methods

### 2.1. Materials

Urea (98%) was produced by PetroChina Ningxia petrochemical company (Ningxia, Chian). NaOH (analytically pure) was produced by the Beijing chemical plant; Phenol (98%) and formaldehyde solution (37–40%) were produced by Xilong Scientific Co., Ltd. (Shantou, China). Soybean meal (industrial-grade, containing 48% of soy protein) was provided by Tianguan Biotech Company Limited in Nanyang of China.

### 2.2. Preparation of PF and SMPF Resins

PF resin was synthesized using our previous work (formaldehyde/phenol molar ratio of 2.2) [1,35]. NaOH solution with a concentration of 50% was used as the catalyst. First, phenol, one third of formaldehyde, and NaOH solution were added into a four-necked flask. Then, the sample solution was heated to 90 °C within 10 min and kept reacting for 60 min. Second, the same formaldehyde and NaOH solution were added and kept reacting for 40 min at 88 °C. Finally, the last of the formaldehyde and NaOH solution were added and reacted for 40 min at 88 °C. After that, the reaction mixture was cooled to 40 °C and the neat PF resin was obtained.

**Scheme 1:**
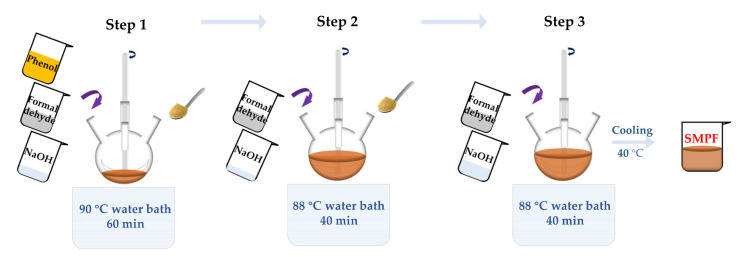
Preparation process of SMPF resin.

SMPF resins were synthesized using the same procedure as PF resin. The process is as shown in Scheme 1. The added amount of soybean meal was in the range of 10–40% by weight of phenol. Firstly, phenol, one-third formaldehyde, half of the soybean meal, and NaOH solution were mixed in a four-necked flask. The mixture was reacted at 90 °C for 60 min. Secondly, another one third of the formaldehyde, the rest of the soybean meal, and one quarter of the NaOH solution (50%) were added, reacting for 40 min at 88 °C. Thirdly, the rest of the formaldehyde and NaOH solution were added (reacting for 40 min at 88 °C). The reaction samples were cooled to 40 °C to obtain the SMPF resins. These SMPF resins were marked as SMPF-1, SMPF-2, SMPF-3, and SMPF-4.

### 2.3. Characterization of the Phenolic Resins

Fourier transform infrared spectroscopy (FTIR) was used to characterize the structure of SM, PF resins, and SMPF resins. All of the samples were freeze-dried at −60 °C for 18 h. Then, these samples were ground into meal in a mortar (200-mesh). Then, the SM, uncured PF, and SMPF resin samples were analyzed with a Nicolet 6700 spectrometer (Nicolet Instrument Corporation, USA) [1]. The PF and SMPF resins were detected using a TA Q2500 (differential scanning calorimeter, TA instruments, USA). The DSC scanning temperature range was 30–180 °C; the heating rate was 10 °C/min [35]. All resin samples were cured at 120 °C for 120 min to measure their thermal stability. About 5–6 mg cured resin samples (200-mesh) were determined using a Q50 analyzer (TA instruments, USA). The conditions of testing were as follows: the temperature range was 30–800 °C, the heating rate was 10 °C/min, and the atmosphere was composed of N_2_. After it was completely dried and cured in a 120 °C oven, the cured resin was broken to obtain a smooth and flat fracture surface. After a gold coating was sprayed on the fracture surface of the sample, it was analyzed and tested using a scanning electron microscope (SU8010, Hitachi, Japan).

For all resins, the performances of gel time, solid content, and pH value were also measured using the method from GB/T 14070-2017 and Li et al. [11]. The residual rate measurement followed the report of Li et al. [36]. Each resin sample test was repeated three times, and the sample’s average value was reported.

### 2.4. Plywood

Three-layer plywood was prepared with poplar veneer, which is 400 mm × 400 mm × 1.5 mm. Both sides of the middle veneer were coated with adhesive, a mixture of phenolic resin, and 15% flour (according to the weight ratio of phenolic resin). About 125–150 g/m^2^ adhesive was coated on the veneer for each side. Firstly, the plywood samples were cold-pressed under 0.5 MPa for 15 min and then hot-pressed at 135 °C under 1.05 MPa for 5 min. The plywood sample was stored at normal temperature for 3 days before testing. Eight plywood specimens were submersed in boiling water for 3 h, then dried at room temperature for 10 min before the testing. The wet shearing strength of plywood was measured following the Chinese national standard GB/T 17657-2013 [35].

## 3. Results and Discussion

### 3.1. Characterization of PF and SMPF Resins

**Figure 1:**
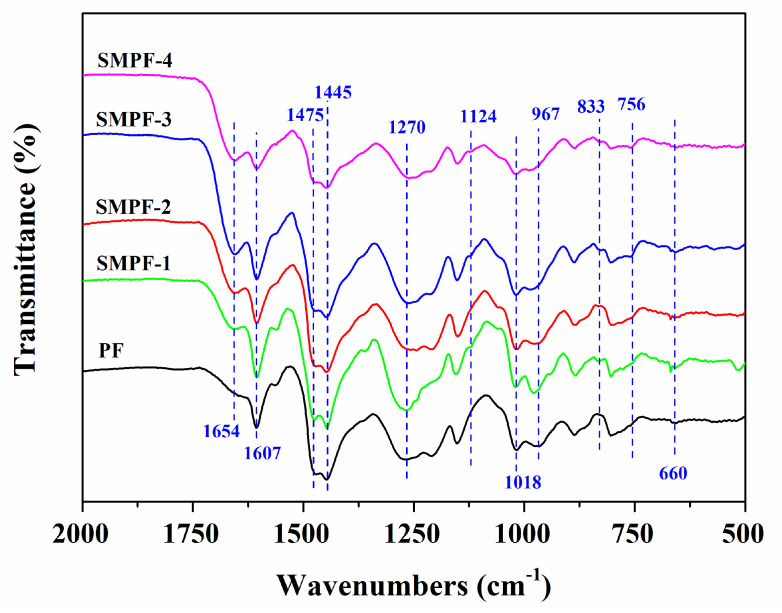
FTIR spectra of the PF and SMPF resins.

As seen in Figure 1, the PF and SMPF resin samples show intense peaks at around 3300–3400 cm^−1^, belonging to the phenolic and aliphatic hydroxyl groups. The absorption peaks at 2900–2860 cm^−1^ can be assigned to the C–H stretching vibration in the methylene groups. The characteristic peaks of aromatic rings in all phenolic resins were observed at 1606, 1457, and 1446 cm^−1^ [13]. The peak at 1244 cm^−1^ was associated with the stretching vibration of C–O in the phenolic hydroxyl groups. The absorption peaks at 829, 757, and 694 cm^−1^ can be attributed to the aromatic C–H [25,29]. In addition, the peak at 978 cm^−1^ was related to vinyl’s C–H stretching vibration [26].

Comparing the FTIR spectra of PF and SMPF resins, we can see that all the samples have similar functional groups, indicating that PF and SMPF have similar molecular structures. However, the FTIR spectra of PF and SMPF resin also have some differences. Compared with PF resin, the spectra of all SMPF resins presented stronger absorption peaks at around 1667 and 1357 cm^−1^ and showed weaker absorption at around 967 cm^−1^. These differences can be attributed to the reaction between hydroxymethyl phenol (HP) and soybean meal, which contained amounts of soy protein. As seen in Figure 1, with the increase in the substitution of phenol and formaldehyde by soybean meal, the peak at 1667 cm^−1^ was stronger. The absorption peak at 1667 cm^−1^ belonged to the C=O stretching vibration of amine groups in the molecular structure of soy protein [37,38], which implied that the higher the phenol substitution by soybean meal, the higher the reaction degree for PF and soy protein [39]. In addition, soybean meal can react with formaldehyde and form hydroxymethylated soybean meal (HSM). The condensation reaction could occur between HSM and HP or HSM and HSM.

### 3.2. Thermal Behaviors of the PF and SMPF Resins

**Figure 2:**
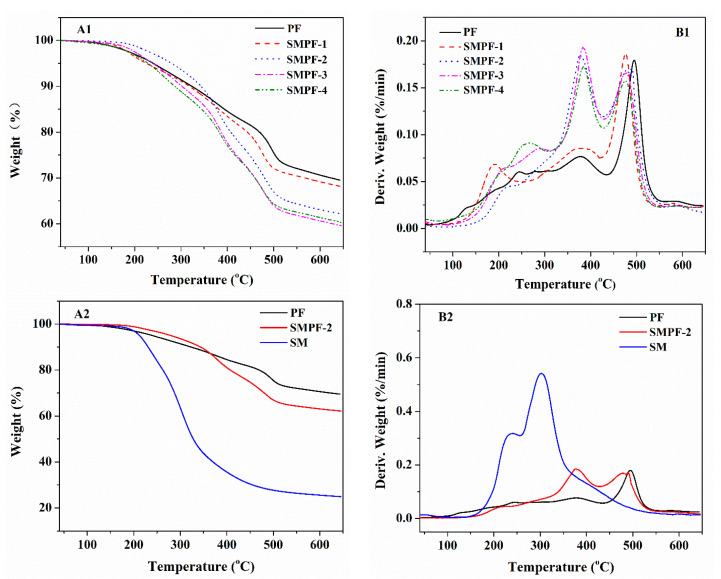
TG and DTG curves of the PF and SMPF resins (**A1**,**B1**); SM, PF, and SMPF-2 resins (**A2**,**B2**).

TGA analysis was performed to investigate the effect of soybean meal on phenolic resin’s thermal stability. The TGA curve results are shown in Figure 2. The weight loss ratio and the peak of the DTG curve (T_max_) could be used to evaluate the thermal stability of resin. The weight loss and T_max_ of different decomposition stages of the resin samples are shown in Table 1.

The phenolic resins generally underwent three main decomposition stages: post-curing, thermal reforming, and ring stripping. According to the curves of the TG and DTG analysis in Figure 2, it can be seen that the main mass losses of all the PF and SMPF resins occurred in the regions of 100–310 °C, 310–430 °C, and 430–550 °C, respectively. In the first region, the mass loss was attributed to the release of water, mainly derived from the cross-linking/condensation reaction between methylol groups [40,41]. For the second region, the mass loss might be due to the decomposition of the methylene bridge bond in phenolic resin. In addition, some studies suggest that the mass loss in this stage might be due to the evaporation of water caused by the condensation reaction between two hydroxyl functional groups and the reaction between methylene and phenolic –OH [42,43]. The aromatic structure was degraded to a carbonaceous structure in the third mass loss region. Moreover, amounts of carbon monoxide and methane were produced from the decomposition of methylene linkage [44,45,46].

The thermal properties of the cured PF and SMPF resins are shown in Table 1. All samples’ weight loss and the T_max_ of different thermal degradation steps are listed. Table 1 shows that the soybean meal significantly affected the phenolic resin’s thermal stability. All the SMPF resins showed lower weight residues at 600 °C than neat PF resin. It was indicated that soybean meal decreased the thermal stability of phenolic resin. This could be attributed to the fact that the SMPF resin contained several carbohydrate compounds, which were derived from soybean meal. These carbohydrate compounds have difficulty reacting with soy protein and hydroxymethyl phenol. Finally, the neat cured PF resin had a tighter network than all the SMPF resins. For all the SMPF resins, SMPF-1 had the highest thermal stability; the total weight loss was 31.86% in the temperature region of 100–550 °C.

### 3.3. The Properties of the PF and SMPF Resins 

The pH value, viscosity, solid content, gel time, and pot life were tested and listed in Table 2 for all resins. As can be seen from Table 2, all the SMPF resins have almost similar pH values and solid contents. Compared with PF resin, the viscosities of all modified resins were increased (from 87 to 1083 mPa‧s). This could be attributed to the amounts of hydrogen bonds in SMPF resins. The soybean meal contained amounts of soy protein and carbohydrate compounds. The molecular chains of these compounds are broken under alkaline conditions, creating a large number of hydrogen bonds [47,48]. The hydrogen bonds in the modified resin systems increased their viscosity. It should be pointed out that the pH values of all phenolic resins were controlled at about 12, which is beneficial to the curing of resin in the process of plywood preparation. Gel time is an important property of phenolic resin, which can be used to evaluate the curing performance of the resin. All the gel times of the SMPF resins were shorter than that of the PF resin. This indicated that the curing rate of these resins is faster than that of PF resin at 150 °C. In order to compare with previous studies and following the requirement of GB/T 14074-2017, 150 °C was selected to measure the gel time of the PF resin [1,35]. The 40% SMPF resin had the maximum viscosity (1083 mPa s). At the same time, its gel time was the shortest, at 305 s. When the amount of soybean meal added is large, during the synthesis process of SMPF resin, under alkaline conditions, soybean meal undergoes degradation, and the peptide chains in it unfold; some break and then copolymerize with phenol and formaldehyde, resulting in a copolymer with a higher molecular weight than pure phenolic resin. In addition, the protein peptide chain structure in soybean meal is prone to hydrogen bonding with the resin system, thereby improving the viscosity of the SMPF resin system. Soybean meal-copolymerization-modified phenolic resin has a high molecular weight, a high viscosity, and is easy to gel, which ultimately shortens the gel time of SPF resin. In addition, it is worth noting that all of the SMPF resins had a long pot life, which benefits their industrial application.

### 3.4. Curing Behavior of the PF and SMPF Resins

**Figure 3:**
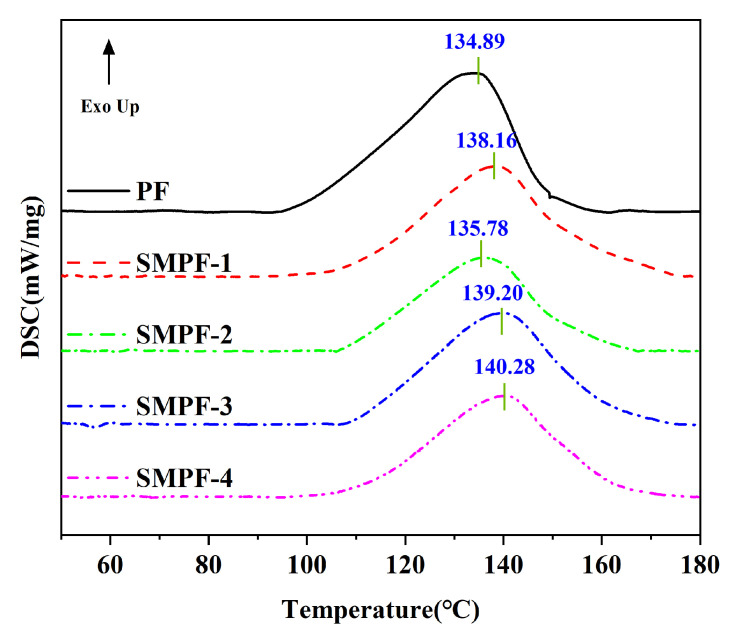
DSC curves of the PF and SMPF resins.

Differential scanning calorimetry analysis is a powerful method to investigate the curing behavior of thermosetting resins [49,50]. This work studied the influence of soybean meal on the curing properties of the PF and SMPF resins. DSC analysis was conducted, and the results were recorded, as shown in Figure 3. The DSC curve (T_peak_) was used to compare the curing properties of different resins.

As seen in Figure 3, the exothermic peaks were observed for all resins. The exothermic peak is attributed to the condensation reaction, which can release energy. In addition, this also indicated that the cross-linking/condensation reaction could have occurred between methylated soybean meal and methylated phenol. From Figure 3, the onset temperature of the PF resin was around 90 °C, but those of the SMPF resins were around 100 °C. Moreover, the exothermic peak temperature of PF, SMPF-1, SMPF-2, SMPF-3, and SMPF-4 were 134.89, 138.16, 135.78, 139.20, and 140.28 °C, respectively. This indicates that the curing temperature of the modified SMPF resins was higher than that of the neat PF resin. Therefore, this suggests that the required temperature of the condensation reaction between methylated soybean protein and methylolphenol is higher than that of the condensation reaction between two methylolphenol groups.

SMPF-4 resin had a maximum peak temperature of 140.28 °C in the DSC curve. SMPF-2 resin had the lowest curing peak temperature (135.78 °C), and its gel time was also relatively short (370 s). Soybean meal contains a large number of starch components. They are difficult to cross-link with hydroxymethyl phenol. The curing peak temperature of SMPF resin is higher than that of PF resin (134.89 °C) in the DSC curves (Figure 3). From Figure 3, it can be seen that the curing peak temperature of modified phenolic resin did not gradually increase with the increase in soybean meal content. Because of the complex composition of soybean meal, the carbohydrates in it had a negative effect on the curing of phenolic resin. There are many factors that affect the peak temperature in resin curing. During the synthesis phase of the resin, reaction temperature, time, pH value, etc. all have an impact on the performance of the resin. Furthermore, the addition of soybean meal affects the difference in the concentration ratio of reactants in the system. Meanwhile, the complexity of soybean meal components (mainly carbohydrates and soy protein) could also affect the curing characteristics of modified resins. In our previous research, we found that an appropriate amount of soy protein can promote the low-temperature solidification of phenolic resin. But when this is excessive, the curing temperature of soybean protein-modified phenolic resin increases with the addition of soybean protein. The starch and protein components in soybean meal compete in the reaction with hydroxymethyl phenol. Ultimately, this affects the curing of soybean meal-modified phenolic resin. The gel time can reflect the curing characteristics of the resin to a certain extent. However, it cannot be guaranteed to fully match the curing characteristics displayed in the DSC test analysis results. For the study of the curing characteristics of phenolic resin, the DSC test analysis results are relatively more accurate and convincing.

### 3.5. Residue Analysis

**Figure 4:**
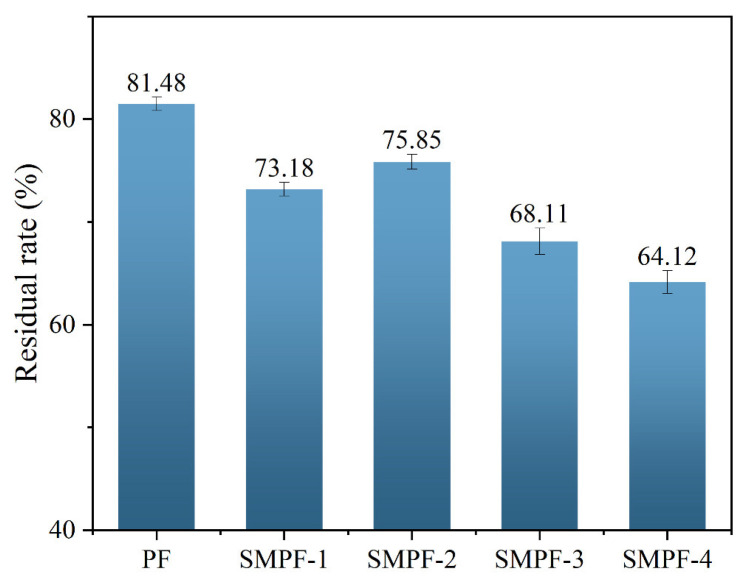
Residual rates of PF and different SMPF resins.

The residual rates of different SMPF resin adhesives are shown in Figure 4. The residual rate of PF resin was 81.48%, higher than those of all the SMPF resin adhesives. The main reason for this is that SM contains a large amount of starch. Starch molecules have hydrophilicity and cannot form a densely cross-linked network structure with PF. Ultimately, the residual rate of SMPF resin is lower than that of PF resin adhesive. The residual rates of SMPF-1 and SMPF-2 were 73.18% and 75.85%, respectively. This is because when appropriate SM is added, the active groups on the molecular protein chain in SM undergo a cross-linking reaction with phenolic resin. The cross-linking density of SMPF resin after curing increases, effectively preventing water from entering the adhesive layer, thereby improving the water resistance of the adhesive. The residual rates of SMPF-3 and SMPF-4 decreased compared with SMPF-1 and SMPF-2, reaching 68.11% and 64.12%. Due to the excessive addition of SM, the starch molecules in SM did not fully participate in the cross-linking and curing reaction of PF resin, thereby reducing the cross-linking density of the adhesive. By adjusting the amount of SM added to balance the optimal reaction ratio between SM and PF, the cross-linking reaction between them can be promoted. When 20% SM is added, the residual rate of SMPF-2 adhesive is relatively high, at 75.85%.

### 3.6. The Wet Shearing Strength of Plywood Using Different SMPF Adhesives

**Figure 5:**
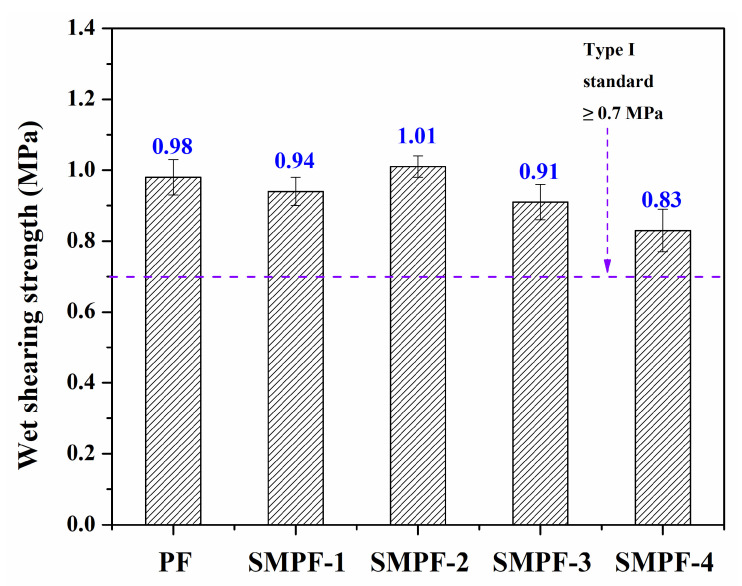
The wet shearing strength of the PF and SMPF resins.

The effect of soybean meal on the performance of plywood was studied. Figure 5 shows the wet shearing strength of all the resins. As seen in Figure 5, all of the plywood bonded with the SMPF resins exhibited excellent water resistance and bonding strength, which could meet the requirement for plywood (the Chinese national standard, type I, ≥0.7 MPa). For all the SMPF resins, the wet shearing strength was lower than that of the PF resin, except for SMPF-2. When 20% soybean meal was added (weight by phenol), the SMPF-2 plywood had the highest bonding strength (1.01 MPa). For the PF resin, although the curing peak temperature was higher than that of the SMPF resin, the bonding strength of the plywood was lower. This might be attributed to the favorable viscosity of the SMPF-2 resin. From Table 2, the SMPF-2 resins showed a suitable viscosity for plywood bonding. This indicates that SMPF-2 has good coating behavior. On the other hand, low viscosity might cause uneven coating issues, which influences PF resin’s performance and reduces its bonding strength [36].

In addition, the tighter network structures formed from the condensation reaction between the hydroxymethyl phenolic structure and soybean meal molecules enhanced the bonding strength and water resistance of the SMPF-2 resin. The wet shearing strength of modified phenolic resin is higher than 0.7 MPa, meeting the relevant requirements of the national standard for Class I plywood (GB/T 17657-2013). This indicates that during the synthesis process of SMPF resin, the protein peptide chain structure and polysaccharide structure in soybean meal undergo a copolymerization reaction with phenol and formaldehyde, forming copolymerized compounds. During the solidification process of SMPF resin, the molecules of soy protein isolate form a cross-linking system with phenolic resin, which has good water resistance and makes the prepared plywood have good water resistance.

### 3.7. SEM Analysis

**Figure 6:**
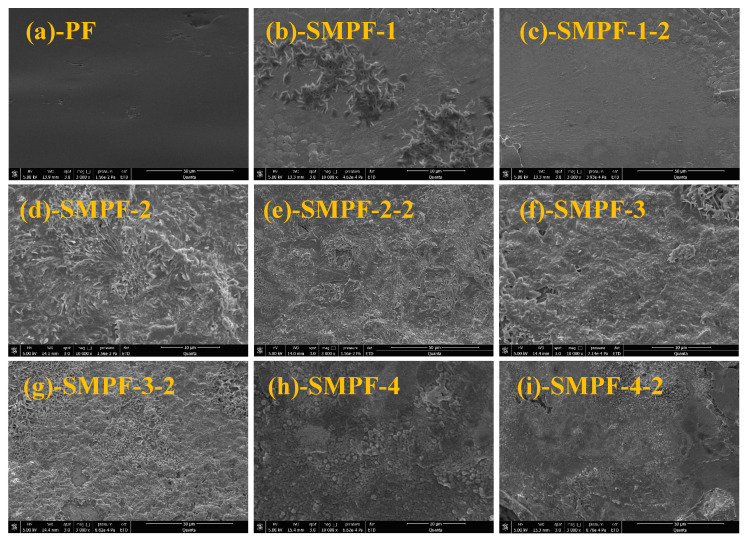
SEM images of the PF and SMPF resins.

Figure 6 shows an SEM image of the fracture morphology of the prepared adhesives. It can be seen that the surface of the PF resin is very uniform and smooth. For the modified SMPF resin with liquefied SM, the cross-sections of all samples become coarse and unsmooth. The main reason for this is that the density of rigid groups in the molecular structure of PF resin is large, and the toughness of the resin after cross-linking and curing is poor, so its cross-section is even and smooth. From Figure 6, it also can be seen that the cross-section of the SMPF resin has undergone significant changes, with a flocculent and irregular surface. The main reason for this is that SM reacts with hydroxymethyl phenol and formaldehyde, forming a cross-linked network structure. SMPF resin has a complex molecular structure and uneven curing compared with PF resin. In addition, the starch components in SM cannot react with formaldehyde and phenol, thus affecting the curing of SMPF resin. The above reasons ultimately resulted in an uneven and irregular cross-section of the cured modified SMPF resin. The cured section of the SMPF-4 resin modified with a 40% SM addition shows obvious cracking, indicating that excessive SM addition increased the brittleness of the modified SMPF resin. The cracking of SMPF-4 resin after curing can reduce its water resistance, resulting in low wet bonding strength (0.83 MPa) and poor water resistance.

## 4. Conclusions

Environmentally friendly phenolic resin with a low cost, excellent water resistance, and excellent bonding strength properties was prepared using soybean meal as a substitution. The FTIR analysis results suggested that a cross-linking reaction occurred between the PF and soybean meal. In addition, soybean meal can react with formaldehyde and form hydroxymethylated soybean meal (HSM). A condensation reaction could occur between HSM and HP or HSM and HSM. The TG analysis results show that the SMPF resins’ degradation increased compared with that of the PF resin. The DSC results indicate that the addition of soybean meal increased the curing temperature of the phenolic resins. The exothermic peak temperatures of PF, SMPF-1, SMPF-2, SMPF-3, and SMPF-4 were 134.89, 138.16, 135.78, 139.20, and 140.28 °C, respectively. This indicates that the curing temperature of the modified SMPF resins is higher than that of the neat PF resin. In addition, this also suggests that the required temperature promoting the condensation reaction between methylated soybean meal and methyl phenol is higher than that of the condensation reaction between two methylolphenol groups. Compared with the other resins, the SMPF-2 resin has the highest wet shearing strength (1.01 MPa). In addition, the plywood performance results also show that the SMPF-2 resins have favorable viscosity (309 mPa‧s), short gel time (370 s), higher residual rates (75.85%), and excellent water resistance, meeting the Chinese national standard for type I plywood. Therefore, the optimized SMPF resin can potentially partially replace PF resin in the wood industry.

## Figures and Tables

**Table 1:** Thermal degradation properties of SMPF resins.

Resins	Step 1 100–310 °C			Step 2 310–430 °C		Step 3 430–550 °C		Total Mass Loss (%)
	T_P1_ (°C)	T_P2_ (°C)	Mass Loss (%)	T_P3_ (°C)	Mass Loss (%)	T_P4_ (°C)	Mass Loss (%)	
PF	245	-	7.89	378	9.05	494	9.72	30.47
SMPF-1	191	-	9.32	384	9.40	475	10.64	31.86
SMPF-2	229	-	6.97	379	15.70	482	12.87	37.80
SMPF-3	226	289	11.37	383	15.52	476	11.92	40.32
SMPF-4	215	265	11.41	384	14.46	475	10.98	39.60

**Table 2:** Characteristics of the PF and SMPF resins.

Resin	pH	Viscosity (mPa‧s)	Solid Content (%)	Gel Time (s)	Pot Life (d)
PF	11.98	87	43.85	571	>30
SMPF-1	12.23	96	39.42	423	>30
SMPF-2	12.25	309	39.78	370	>30
SMPF-3	12.17	394	39.64	406	>30
SMPF-4	12.24	1083	39.52	305	>30

## Data Availability

Not applicable.
